# Reviewer acknowledgement 2014

**DOI:** 10.1186/s13005-015-0059-8

**Published:** 2015-05-09

**Authors:** Thomas Stamm, Ulrich Meyer, Hans Peter Wiesmann

**Affiliations:** University of Münster, Münster, Germany; University of Düsseldorf, Düsseldorf, Germany; Technische Universität Dresden, Dresden, Germany

## Abstract

The Editors of *Head & Face Medicine* would like to thank all our reviewers who have contributed to the journal in Volume 10 (2014).

**Ahmad Abdelkarim**

USA

**Lucas Abreu**

Brazil

**Jose Manuel Almerich**

Spain

**Nivaldo Alonso**

Brazil

**Fernando Antonini**

Brazil

**Antonio Antunes**

Brazil

**Gururaj Arakeri**

India

**Erica Avila**

USA

**Ashraf Ayoub**

United Kingdom

**Tomislav Badel**

Croatia

**Hyoung-Seon Baik**

South Korea

**Paula Bavia**

Brazil

**Boulos Bechara**

USA

**Paola Beltri**

Spain

**Darpan Bhargava**

India

**Dirk Bister**

United Kingdom

**Niko Bock**

Germany

**Steve Bowman**

USA

**Sandra Bussadori**

Brazil

**Halil Çagatay**

Turkey

**Patrícia Caldeira**

Brazil

**Maria Clotilde Carra**

USA

**Michele Cassetta**

Italy

**Cinzia Cavestro**

Italy

**Murat Cehreli**

Turkey

**Luzi Cesare**

Italy

**Indranil Chakrabarti**

India

**Zhengxi Chen**

China

**Wah Cheuk**

Hong Kong

**Daniel Cheuk**

Hong Kong

**Sung-Hwan Choi**

South Korea

**Monique Chouvin**

France

**Tam-Lin Chow**

Hong Kong

**Fernando Coelho**

Brazil

**Edouard Coeugniet**

Canada

**Hakan Çolak**

Iraq

**Harun Çöloglu**

Turkey

**Maria S Commisso**

France

**Everton da Rosa**

Brazil

**Emad Daif**

Egypt

**Domenico Dalessandri**

Italy

**Till Dammaschke**

Germany

**Gholamreza Danesh**

Germany

**Cees de Baat**

The Netherlands

**Julio Cesar de Oliveira**

Brazil

**Giacomo Del Corso**

Italy

**Alexander Delides**

Greece

**Cagri Delilbasi**

Turkey

**Nermin Demirkol**

Turkey

**Ömür Dereci**

Turkey

**Kartik Dholakia**

India

**Ilze Dobele**

Latvia

**Angela Dominguez**

Colombia

**Umal Doshi**

India

**Amit Nandan Dhar Dwivedi**

India

**Ali Farahani**

United Kingdom

**Imre Fejes**

Hungary

**Andreas Filippi**

Switzerland

**Roman Fischbach**

Germany

**Roberto Flores**

USA

**Claire Forbes-Haley**

United Kingdom

**Ana Lúcia Franco**

Brazil

**Bernhard Frerich**

Germany

**Zhen Fu**

China

**Patrick Garvey**

USA

**Volker Gassling**

Germany

**Olaide S Gbadebo**

Nigeria

**Nils-Claudius Gellrich**

Germany

**Negin Ghaffari**

Iran

**Nikolaos Gkantidis**

Switzerland

**Agnieszka Gniadek**

Poland

**Neeraja Gokhale**

India

**Michel Goldberg**

France

**Nathan Gonik**

USA

**Dan Grauer**

USA

**Christian Gueldner**

Germany

**Maria do Carmo Guimarães**

Brazil

**Orlando Guntinas-Lichius**

Germany

**Hongqiang Guo**

USA

**Deepak Gupta**

India

**Samson Gwer**

Kenya

**Urban Hagg**

China

**Tadasu Haketa**

Japan

**Jörg Günter Kurt Handschel**

Germany

**Shin Hatou**

Japan

**Rashmi Hegde**

India

**Samuel Ho**

Singapore

**Raphael Hoehr**

Germany

**Ariane Hohoff**

Germany

**Christof Holberg**

Germany

**Luisa Hopker**

Brazil

**Luis Huanca Ghislanzoni**

Italy

**Ren-Yeong Huang**

Taiwan

**Christopher Hughes**

USA

**Mitsuyoshi Iino**

Japan

**Gianluca Ingrosso**

Italy

**Belma Isik Aslan**

Turkey

**Abdolreza Jamilian**

Iran

**Ashok Jena**

India

**Asbjorn Jokstad**

Norway

**Christof Joss**

Switzerland

**Susanne Jung**

Germany

**Graça Justo**

Brazil

**Yuhji Kabasawa**

Japan

**Jenny Kallunki**

Sweden

**Spyridon Karras**

Greece

**Adrian Kasaj**

Germany

**Naoki Kato**

Japan

**Vivekanand Kattimani**

India

**Joseph Katz**

USA

**Chung How Kau**

USA

**Richa Khanna**

India

**Reuben Kim**

USA

**Christian Kirschneck**

Germany

**Johannes Kleinheinz**

Germany

**Tejs Klug**

Denmark

**Michael Knoesel**

Germany

**Felix Koch**

Germany

**Steffen Koerdt**

Germany

**Leonardo Koerich de Paula**

USA

**Yichun Kong**

China

**Bernd Koos**

Germany

**Stefan Kopp**

Germany

**Heike Korbmacher-Steiner**

Germany

**Jolanta Kostrzewa-Janicka**

Poland

**Elena Krieger**

Germany

**Branko Kristo**

Bosnia and Herzegovina

**Ismail Küçüker**

Turkey

**Francisco Larrosa**

Spain

**Guenter Lauer**

Germany

**Andrezza Lauria**

Brazil

**Xavier León**

Spain

**Ilya Leyngold**

USA

**Tang Lijun**

China

**Carsten Lippold**

Germany

**Jörg Lisson**

Germany

**Anders Löfqvist**

USA

**Heather Logan**

Canada

**Dominic Lu**

USA

**Rong Lu**

China

**Muhammad Haris Lucky**

Pakistan

**Björn Ludiwg**

Germany

**Christopher J Lux**

Germany

**Caoimhin Mac Giolla Phadraig**

Ireland

**Juliana Marotti**

Germany

**Jorge Martins**

Portugal

**Ranieri Martuscelli**

Italy

**Cinzia Maspero**

Italy

**Astuji Matsuyama**

Japan

**Carly McKenzIe**

USA

**Bodo Melnik**

Germany

**Maria Elissavet Metska**

The Netherlands

**Philipp Metzler**

Switzerland

**Shaza Mohammad**

Egypt

**Hamid Reza Mollaei**

Iran

**Paul Monsarrat**

France

**Christopher Moskaluk**

USA

**Alexandre Motta**

Brazil

**Mario Muñoz Guerra**

Spain

**Toshitaka Nagao**

Japan

**Tomohisa Nagasao**

Japan

**Tetsuji Nakamoto**

Japan

**Teresa Neuhann**

Germany

**Sizakele Ngwenya**

South Africa

**Yousef Wirenfeldt Nielsen**

Denmark

**Manuel Nienkemper**

Germany

**Jun Nihara**

Japan

**Lluís Nisa**

Switzerland

**George Obeid**

USA

**Kent Ochiai**

USA

**Seigo Ohba**

Japan

**Siti Adibah Othman**

Malaysia

**Eleni Panou**

Greece

**Jae Park**

USA

**Eudoxie Pepelassi**

Greece

**Letizia Perillo**

Italy

**Jerome Philip**

United Kingdom

**Francesco Pieri**

Italy

**József Piffkó**

Hungary

**Anne-Lise Poirrier**

Belgium

**Michal Polguj**

Poland

**Claus Pototschnig**

Austria

**Amaury Pozos**

Mexico

**Gabriela Prado**

Brazil

**Peter Proff**

Germany

**Vahid Rakhshan**

Iran

**Ingrid Rudzki**

Germany

**Maria Sabbatini**

USA

**Hans-Guenter Schaller**

Germany

**Nicole Scheffler**

USA

**Harry C Schwartz**

USA

**Robin Seeberger**

Germany

**Firat Selvi**

Turkey

**Michael Shterenshis**

Israel

**Armando Silvestrini Biavati**

Italy

**Kunwarjeet Singh**

India

**Rameshwari Singhal**

India

**Aaron Smith**

USA

**Janet Southerland**

USA

**Stjepan Spalj**

Croatia

**Joerg Tchorz**

Germany

**Tiziano Testori**

Italy

**Afshin Teymoortash**

Germany

**Trevor Thompson**

United Kingdom

**Tolga Topcuoglu**

Turkey

**Konstantinos Tosios**

Greece

**Giedre Trakiniene**

Lithuania

**Matthias Troeltzsch**

Germany

**Koichiro Ueki**

Japan

**Cem Ungor**

Turkey

**Vinay Kumar**

India

**Gabor Varga**

Hungary

**Tomaz Velnar**

Slovenia

**Joan Viciano**

Italy

**Giovanna Viticchi**

Italy

**Pawanjit Singh Walia**

India

**Toru Watanabe**

Japan

**Ashley Weaver**

USA

**Dirk Wiechmann**

Germany

**Yunong Wu**

China

**Rong-hsin Yang**

Taiwan

**Nuray Yilmaz Altintas**

Turkey

**Abbas Zaher**

Egypt

**Xianglong Zeng**

China

